# Axonal Projection Patterns of the Dorsal Interneuron Populations in the Embryonic Hindbrain

**DOI:** 10.3389/fnana.2021.793161

**Published:** 2021-12-24

**Authors:** Dana Hirsch, Ayelet Kohl, Yuan Wang, Dalit Sela-Donenfeld

**Affiliations:** ^1^Koret School of Veterinary Medicine, The Robert H. Smith Faculty of Agriculture, Food and Environment, The Hebrew University of Jerusalem, Rehovot, Israel; ^2^Department of Veterinary Resources, Weizmann Institute of Science, Rehovot, Israel; ^3^Department of Biomedical Sciences, Program in Neuroscience, College of Medicine, Florida State University, Tallahassee, FL, United States

**Keywords:** hindbrain, rhombomere, dorsal interneurons, rhombic lip, axonal growth

## Abstract

Unraveling the inner workings of neural circuits entails understanding the cellular origin and axonal pathfinding of various neuronal groups during development. In the embryonic hindbrain, different subtypes of dorsal interneurons (dINs) evolve along the dorsal-ventral (DV) axis of rhombomeres and are imperative for the assembly of central brainstem circuits. dINs are divided into two classes, class A and class B, each containing four neuronal subgroups (dA1-4 and dB1-4) that are born in well-defined DV positions. While all interneurons belonging to class A express the transcription factor Olig3 and become excitatory, all class B interneurons express the transcription factor Lbx1 but are diverse in their excitatory or inhibitory fate. Moreover, within every class, each interneuron subtype displays its own specification genes and axonal projection patterns which are required to govern the stage-by-stage assembly of their connectivity toward their target sites. Remarkably, despite the similar genetic landmark of each dINs subgroup along the anterior-posterior (AP) axis of the hindbrain, genetic fate maps of some dA/dB neuronal subtypes uncovered their contribution to different nuclei centers in relation to their rhombomeric origin. Thus, DV and AP positional information has to be orchestrated in each dA/dB subpopulation to form distinct neuronal circuits in the hindbrain. Over the span of several decades, different axonal routes have been well-documented to dynamically emerge and grow throughout the hindbrain DV and AP positions. Yet, the genetic link between these distinct axonal bundles and their neuronal origin is not fully clear. In this study, we reviewed the available data regarding the association between the specification of early-born dorsal interneuron subpopulations in the hindbrain and their axonal circuitry development and fate, as well as the present existing knowledge on molecular effectors underlying the process of axonal growth.

## Introduction

The vertebrate central nervous system (CNS) is composed of a vast array of neuronal circuits that are assembled in a stepwise manner to give rise to the enormous diversity of cells and functions. Cell fate acquisition, neural cell migration, and axonal projections are all initiated in the developing neural tube and give rise to the neuronal networks of genetically defined neurons that are interconnected within the CNS, as well as with afferent/efferent connections with peripheral targets. It is only with the advent of molecular techniques of lineage tracing, designated gene mutations, and enhanced developmental analyses that have allowed the emerging understanding of the details of neural connectivity assembly. Although many studies uncovered genes that regulate different aspects of the multi-event process that spans from neural specification to circuit formation, there is still missing knowledge regarding how these complex mechanisms are orchestrated to give rise to functional networks, and what goes wrong in neurodevelopmental disorders.

The early development of the CNS starts with a series of swellings followed by elementary division into the forebrain, midbrain, and hindbrain. The hindbrain, which is a highly conserved region across vertebrates, has been traditionally subdivided into the pons, medulla oblongata, and cerebellum, which together compose the brainstem. Notably, a more accurate subdivision of the hindbrain into the prepontine, pontine, retropontine, and medullary sub-domains has been recommended based on cell fate mapping and gene expression analyses (Watson et al., 2019). Positioned between the spinal cord and upper brain, the hindbrain serves as a key relay-hub linking the lower and upper parts of the CNS as well as the cranial peripheral nervous system (PNS) *via* multiple circuits that regulate vital functions such as breathing, fine-tuning movement, blood pressure adjustment, auditory and vestibular sensations and facial movement (Joyner and Zervas, 2006; Stiles and Jernigan, 2010; Champagnat et al., 2011; Nothwang et al., 2015; Hernandez-Miranda et al., 2017; Glover, 2020).

**FIGURE 1:**
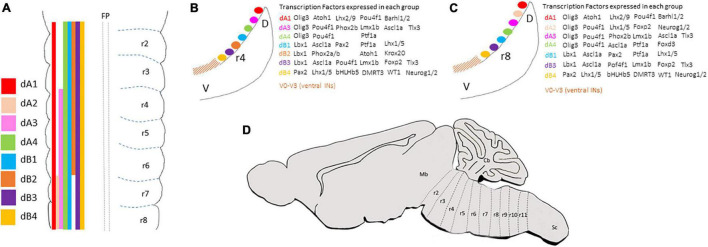
**(A)** Summary of the anterior-posterior distribution of dA and dB subclasses along the overt hindbrain rhombomeres (as classified in Lumsden and Krumlauf, 1996). **(B,C)** Schematic transverse sections taken from rhombomere 4 **(B)** or rhombomere 8 **(C)** levels of E3 chick embryo to show the dorsal-ventral distribution of dA and dB subclasses and the combination of transcription factors expressed in each subclass. **(D)** A schematic sagittal section representing the classification of hindbrain segments into 12 rhombomeres based on fate map analyses (as classified in Marín et al., 2008; Tomás-Roca et al., 2016). Cb, cerebellum; D, dorsal; V, ventral; Mb, midbrain; r, rhombomere; Sc, spinal cord.

A landmark in hindbrain ontogeny is its segmentation into 7/8 overt units, termed rhombomeres (identified as r1–r8, from anterior to posterior), along the anterior-posterior (AP) extent of the hindbrain anlage (Figure 1A) (Lumsden and Krumlauf, 1996; Moens and Prince, 2002). A more updated classification lists 12 rhombomeres (r0–r11), some of which (r1–r7) are anatomically delimited by constrictive transverse boundaries, while others (r0, r8–r11), are delimited by molecular and cell lineage parameters and are known as crypto-rhombomeres (also called pseudo-rhombomeres) (Figure 1D). The anterior and posterior-most crypto-rhombomeres define the borders between the hindbrain and the midbrain and spinal-cord (Marin et al., 1995; Cambronero and Puelles, 2000; Marín et al., 2008; Puelles et al., 2013; Soares et al., 2013; Tomás-Roca et al., 2016; Watson et al., 2017).

In all vertebrates, hindbrain compartmentalization is controlled by combinatorial expression and activity of multiple families of transcription and signaling factors across each rhombomere, leading to the generation of distinct cell lineage compartments (Kiecker and Lumsden, 2005; Weisinger et al., 2008, 2010; Chambers et al., 2009; Kayam et al., 2013; Frank and Sela-Donenfeld, 2018; Parker and Krumlauf, 2020). The identity of individual rhombomeres instructs the neuronal differentiation plan in the hindbrain which is manifested in the migration of neurons, axons, and neural crest cells and the generation of different motor or sensory nuclei along defined dorsoventral (DV) and AP positions (Lumsden and Keynes, 1989; Marín and Puelles, 1995; Cambronero and Puelles, 2000; Trainor and Krumlauf, 2001; Briscoe and Wilkinson, 2004; Guthrie, 2007; Marín et al., 2008; Narita and Rijli, 2009; Puelles et al., 2013; Tomás-Roca et al., 2016; Di Bonito and Studer, 2017). Based on the pioneer insights of Wilhelm His from the 19th century, the dorsolateral margin of the longitudinal hindbrain has been defined as the rhombic lip (RL), which has been found to serve as a source of a number of hindbrain neuron populations that are generated through tangential migrations of neuroblasts which delaminate from the RL (Glover et al., 2018). These different neuronal subtypes were found to depend on their rhombomeric origin; in r1, the most dorsal part of the RL contributes a large migratory cell population that forms the external and internal granular layers of the cerebellum (Ben-Arie et al., 1997; Wingate and Hatten, 1999; Köster and Fraser, 2001; Machold and Fishell, 2005). In r2–r6, the same RL domain generates auditory and vestibular nuclei, through which information is processed and relayed to the upper brain and spinal cord, whereas, in r6–r8, it will give rise to multiple pre-cerebellar nuclei which relay peripheral sensation to the cerebellum through mossy fiber neurons (Altman and Bayer, 1980, 1987a,b,c,d; Rubel and Parks, 1988; Cambronero and Puelles, 2000; Rodriguez and Dymecki, 2000; Bermingham et al., 2001; Díaz et al., 2003; Ryugo and Parks, 2003; Pasqualetti et al., 2007; Hoshino et al., 2013; Kratochwil et al., 2017; Díaz and Puelles, 2019; Elliott et al., 2021). Similarly, different types of respiratory and viscerosensory nuclei are suggested to be born from more ventral positions of the RL at distinct axial levels, such as the parabrachial and Kölliker-Fuse nuclei that derive from r1, the A5 and intertrigeminal region that derives from r4–r6, the PreBötzinger complex and retrotrapezoid nucleus (RTN) that derive from r3/r5, and the nucleus tractus solitaries that is thought to derive from more posterior rhombomeres (Qian et al., 2001; Gray, 2008). The hindbrain is also divided along its DV axis into a basal and alar plate, at which discrete neuronal progenitors become specified and differentiate in distinct longitudinal DV locations that are uniform along with the hindbrain (Figures 1A–C) (Marin et al., 1995; Shoji et al., 1996; Cambronero and Puelles, 2000; Schubert et al., 2001; Wang et al., 2005; Zijing et al., 2008; Storm et al., 2009; Hernandez-Miranda et al., 2017). The neural diversity along the AP and DV axes is critical for the correct elaboration of functional circuits that shape the adult brainstem.

Investigation of the neuronal patterns along the dorsal hindbrain has identified 6–8 progenitor domains that are born in designated positions in some or all rhombomeres (Figure 1A). In contrast, it is not yet clear whether the same set of progenitor domains exist at the rostral-most hindbrain levels (r0 and r1), which are largely patterned by the isthmic organizer. Notably, their precise DV distributions continue further caudally to the spinal cord. Altogether, these progenitors will give rise to second-order interneurons that act as first central relay stations for sensory-motor connections, which intervene in reflex arcs or are largely conveyed from the spinal cord and PNS to upper brain centers or to the spinal cord (Logan et al., 1998; Maklad and Fritzsch, 2003; Ryugo and Parks, 2003; Landsberg et al., 2005; Sieber et al., 2007; Rose et al., 2009a,b; Storm et al., 2009). These hindbrain dorsal interneurons (termed here dINs) are divided into class A (dA) and B (dB) neurons based on their DV positions; those who arise in the dorsal microzones of the hindbrain are classified as class A and express the bHLH transcription factor Olig3, while those born more ventrally in the alar plate and express the Ladybird Homeobox transcription factor Lbx1 are referred as class B (Figure 1A) (Gross et al., 2002; Müller et al., 2002, 2005; Zijing et al., 2008; Hoshino, 2012; Puelles, 2013). Each of the two groups is further subdivided along the DV axis based on the expression of unique sets of specification and differentiation genes in various neuroepithelial microzones, that also differ along the ventricular/mantle zone, reflecting the differential progenitor origin and molecular profile of each neuron (Figures 1B,C) (Wang et al., 2005; Fujiyama et al., 2009; Kohl et al., 2012; Gray, 2013; Hernandez-Miranda et al., 2017).

The specification of the different dA/dB subclasses requires coordinated signaling cues that arise from the roof and floor plate (FP) (i.e., BMP, Wnt, and SHH), and provide positional information which leads to the birth of individual neuronal fates (Liem et al., 1997; Lee et al., 1998, 2000; Briscoe et al., 1999; Gaufo et al., 2000; Vogel-Höpker and Rohrer, 2002; Müller et al., 2005; Storm et al., 2009; Tilleman et al., 2010; Moreno-Bravo et al., 2014; Lihua et al., 2019). The mechanisms by which these morphogens act to pattern hindbrain dINs are not fully understood and will not be discussed further in this review.

Following the differentiation of neurons, they start migrating to their final destinations in the mantle layer as well as to project axons that extend toward their target sites in a stepwise manner, under the control of guidance cues along their pathways (Tessier-Lavigne and Goodman, 1996; Chédotal and Richards, 2010). In the developing hindbrain, axonal growth initiates at particular DV positions within each rhombomere and projects into defined commissural and ipsilateral tracts. Upon the completion of the axonal circuit, these tracts will project sensory information from the periphery, spinal cord, and brainstem to higher brain centers, as well as transmit motor commands from the brain to the spinal cord (Rubel and Parks, 1975, 1988; Díaz et al., 1998; Howell et al., 2007; Renier et al., 2010; Kalinovsky et al., 2011; Di Bonito et al., 2017). Over the past 50 years, classical labeling techniques have thoroughly mapped multiple axonal tracts in the hindbrain. Yet, their association to specific dA/dB sub-populations was missing, as most of these studies preceded the development of genetic tools to fate map individual cell groups. Subsequently, the contribution of different dA/dB neural precursors into neuronal populations of different brainstem nuclei has begun to be recognized, as well as the identification of genetically identified tracts that emerge from these centers. Nevertheless, the fate and axonal routes of some of these subgroups are not fully revealed, nor was the delineation of the entire axonogenesis of individual dA/dB subpopulations, from soon after their differentiation until their axons terminate at their targets.

This review aims to cover the gap between the vast knowledge on hindbrain nuclei projections and their association to specific dA/dB INs along the hindbrain AP axis. We will not discuss r0, r1, and their contribution to the isthmus and cerebellum, and mainly concentrate on r2–r7/8, the rhombomeric units which are morphologically evident and coincide in the two rhombomere-classification systems in use (Lumsden and Krumlauf, 1996; Tomás-Roca et al., 2016). Moreover, past and current knowledge regarding molecular cues that govern the axonal growth of dA/dB neurons will also be presented.

### Class A: dA1 Neurons

#### Specification and Fate

This neuronal subpopulation is positioned in the dorsal most portion of the hindbrain RL, bordering the expanded roof plate of the fourth ventricle (Figure 1A). The specification of this excitatory/glutamatergic neuronal population is dependent on the co-expression of Olig3 and the basic helix-loop-helix (bHLH) transcription factor Atonal homolog 1 (Atoh1) in the ventricular zone. A combinatorial expression of the LIM-homeodomain (LIM-HD) transcription factors Lhx2 and Lhx9, the Barh like homeobox transcription factor Barhl1/2, and the POU domain class 4 transcription factor 1 Pou4f1 accompanies dA1 neural differentiation and migration (Figures 1B,C) (Helms and Johnson, 2003; Wang et al., 2005; Kohl et al., 2012; Hernandez-Miranda et al., 2017). Moreover, the two Lim homeobox transcription factors, Lmx1a and Lmx1b, which are expressed in the roof-plate dorsal to dA1 group, are necessary for their specification (Elliott et al., 2021). Extensive fate map studies in mouse and chick hindbrains, together with the generation of knock-in and knock-out mice lines revealed a wealth of derivatives that originate from dA1 INs, depending upon their rhombomeric origin and time of birth; At r1, dA1 neurons give rise to cerebellar granule cells whereas at r2–r8 they give rise to excitatory neurons that assemble various sorts of brainstem nuclei, with some divergence in their rhombomeric origin in avian and mammalians (Farago et al., 2006; Nothwang, 2016; Lipovsek and Wingate, 2018). These include Atoh1^+^ neurons in several nuclei subtypes in the auditory system, such as in the ventral and dorsal cochlear nuclei (VCN, DCN) and the superior olivary nuclei (SON), Atoh1^+^ neurons in the spinal, medial, and lateral vestibular nuclei (Sp5, MVN, and LVN) and in the vestibular nucleus X, Atoh1^+^ neurons in the main and descending sensory spinal trigeminal nuclei and Atoh1^+^ neurons which establish the various precerebellar nuclei (PCNs), which include the pontine gray nuclei (PGN), reticulotegmental nucleus, lateral reticular nucleus (LRN), and external cuneate nucleus (ECN) (Ben-Arie et al., 1997; Bermingham et al., 2001; Landsberg et al., 2005; Machold and Fishell, 2005; Wang et al., 2005, 2020; Farago et al., 2006; Kawauchi et al., 2006; Zijing et al., 2008; Maricich et al., 2009; Ray and Dymecki, 2009; Rose et al., 2009a,b; Machold et al., 2011; Fu et al., 2013; Hoshino et al., 2013; Lipovsek and Wingate, 2018; Elliott et al., 2021). Interestingly, the specification of the choroid plexus is also dependent on the presence of the adjacent dA1 neurons (Elliott et al., 2021). All of the above-mentioned nuclei centers fail to be generated normally in Atoh1*-*null mice, as well as in mice lacking the roof-plate Lim-HD proteins Lmx1A/B, which show various neurodevelopmental defects and die at birth (Mishima et al., 2009; Rose et al., 2009a,b; van der Heijden and Zoghbi, 2018; Chizhikov et al., 2021). Despite this extensive knowledge, the mechanisms that drive dA1/Atoh1^+^ dINs to give rise to such a remarkable wealth of neuronal fates are only partially clear.

#### dA1 Axonal Projections

**FIGURE 2:**
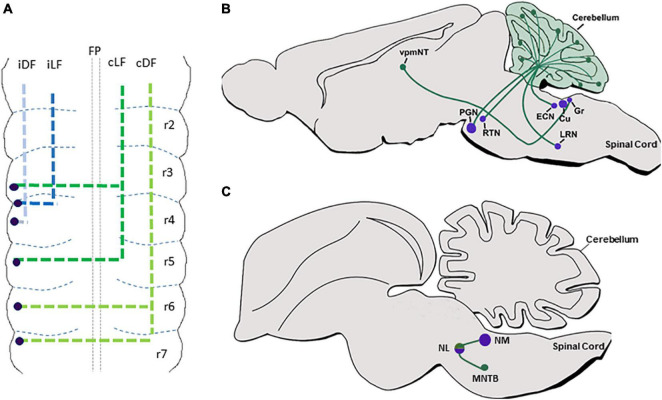
dA1 axonal projections. **(A)** A summary of dA1 axonal projections as seen in a flat-mount view of E6.5 chick embryonic hindbrain. dA1 cell bodies are shown in blue dots. Each axonal tract is shown in a different color and refers to a distinct funiculus. **(B)** A schematic sagittal section of the main pre-cerebellar axonal circuits of the dA1 subclass. Different dA1-derived pre-cerebellar nuclei (purple circles) are shown to project axons (green lines) to the cerebellar granular layer or the thalamus (green circles). **(C)** A schematic sagittal section of the main auditory axonal circuit of the dA1 subclass in the chick hindbrain. dA1-derived NM and NL centers (purple circles) are shown to project axons (green lines) to local auditory nuclei (green circles). FP, floor plate; iDF, ipsi dorsal funiculus; iLF, ipsilateral funiculus; cLF, contralateral lateral funiculus; cDF, contralateral dorsal funiculus; PGN, pontine gray nucleus; RTN, reticulotegmental nucleus; LRN, lateral reticular nucleus; ECN, external cuneate nucleus; Cu, cuneate; Gr, gracile; vpmNT, the ventral posteromedial nucleus of the thalamus; NM, nucleus magnocellularis; NL, nucleus laminaris; MNTB, medial nucleus of the trapezoid body.

Classical axonal labeling approaches in the chick embryonic hindbrain uncovered multiple ascending and descending tracts that arose from ipsilateral or contralateral neurons (Lumsden and Keynes, 1989; Marín and Puelles, 1995; Díaz et al., 2003; Guthrie, 2007). For example, DiI labeling of caudal dorsal hindbrain commissures identified them as formed by second-order vestibular neurons (cC-VC), that project axons which turn into the Dorsal Funiculus (DF) (Díaz et al., 1998; Zhu et al., 2006). Tracing the projections of the cochlear nuclei (CN) showed that the mammalian VCN, or its avian homolog nucleus magnocellularis (NM), project ipsi and contralateral axons to the mammalian SON/avian nucleus laminaris (NL). This symmetrical connectivity results in bilateral excitatory input to the Medial Superior Olivary (MSO) center in the SON in mammalians, or to the NL in avians, that in turn project through ipsi and contralateral lateral lemniscus to midbrain auditory centers (Rubel and Parks, 1988; Kil et al., 1995; Moore, 2000; Carr and Soares, 2002; Seidl et al., 2013). Moreover, labeling of the caudal rodent brainstem revealed ipsi and contralateral projections from different PCN to the cerebellum *via* Mossy Fiber (MF) tracts (Gerrits et al., 1984; Altman and Bayer, 1987a,c,d; Bourrat and Sotelo, 1990; Cicirata et al., 2005; Okada et al., 2007). Furthermore, labeling of excitatory axons of the MVN demonstrated their projections to other brainstem vestibular nuclei together with their projections to the cerebellum as mossy fibers (Ando et al., 2020). Based on the neuronal cell body positions of these axons, it is likely that most of those tracts originate from dA1 neurons at the dorsal RL (excluding Atoh1^+^ neurons in various VN as their axonal projections and later fates are still vague). However, only with the development of genetic tools that utilized Atho1-enhancer elements to label neuronal precursors in the dorsal RL, these trajectories could be attributed to the dA1 subpopulation (Helms and Johnson, 1998; Wang et al., 2005; Farago et al., 2006; Okada et al., 2007; DiPietrantonio and Dymecki, 2009; Rose et al., 2009a,b; Kohl et al., 2012). Subtractive fate map approaches that combined targeting of Atho1^+^ neurons under rhombomere-specific regulatory elements further enabled to reveal the rhombomeric origin of various dA1-derived nuclei (Farago et al., 2006; Maricich et al., 2009; Lipovsek and Wingate, 2018). As such, dA1/Atoh1^+^ neurons that emerge from r2–r5 in mammalians or r5–r8 in avian, and contribute excitatory neurons to the mammalian VCN/avian NM were found to extend ipsilateral and contralateral projections to the mammalian MSO/avian NL (Figure 2C), while those arising from r6–r8 give rise to multiple PCN and extend ipsi and contralateral axons to the granular layer of the cerebellum as MFs (Figure 2B).

Despite this extensive knowledge, data regarding the precise en-route axonal patterns of individual dA1-neuronal subgroups was limited, mostly since dA1-derived axons were largely traced at stages when their soma have already settled at their final nuclear destination. Moreover, since germ-line transgenesis resulted in the labeling of all Atoh1^+^ lineages on both sides of the hindbrain, the ability to trace unilateral projections at subsequent stages was compromised. In a series of studies in the chick hindbrain, we aimed at tracing the sequential axonal growth of dA1 INs at multiple stages, using unilateral electroporation of plasmids encoding dA1-specific enhancers upstream of Cre recombinase together with nuclear, cytoplasmic, or synaptic GFP reporters. Hindbrains were electroporated at stages when dA1/Atoh1^+^ cells are specified and their axons were traced for 2–16 days (Figure 2A) (Kohl et al., 2012, 2013; Hadas et al., 2014). Axons were found to project into the ipsi and contralateral DF and lateral funiculus (LF). Yet, axons that originated from r6–r7 were found to project along with the DF toward the cerebellum and midbrain, whereas those originating from r3–r5 ascended in the LF in a less-tight bundle toward the midbrain (Figure 2A). Pre-synaptic connections of axons from r6–r7 levels terminated in the cerebellar outer and inner granular layers (Figure 2B) (Kohl et al., 2012). These results expanded previous studies performed on post-natal pre-cerebellar-cerebellar mossy fiber circuitry (Marín and Puelles, 1995; Díaz et al., 2003; Wang et al., 2005; Fujiyama et al., 2009; Rose et al., 2009a,b; Kohl et al., 2012, 2013, 2015), by tracing the gradual assembly of these circuits (Figure 2). Using this approach, we have also recently decoded the gradual axonal circuit formation of the binaural auditory system. In this system, dA1 neurons from the avian NM/mammalian anteroventral cochlear nucleus (AVCN) receive temporally locked excitation from the auditory nerve, and in turn, send bilaterally segregated signals to the avian NL/mammalian medial superior olive (MSO), (Cramer et al., 2000; Ryugo and Parks, 2003). The bipolar neurons in the NL/MSO are specialized to compute interaural time differences which is critical for sound localization and segregation (Overholt et al., 1992; Nothwang, 2016). Although the anatomy and physiology of this circuitry are well known, its stage-by-stage assembly was obscure. dA1-specific reporter plasmids were introduced into dA1/NM precursors at r5 and their axons could be traced to gradually exhibit the characterized pattern of NM-NL projection (Figure 2C). This selective dissection allowed us to demonstrate a dA1 subtype-specific, longitudinal assessment of axonal events throughout hindbrain development, from E2.5 to E19 (Wang et al., 2020).

### Class A: dA2 Neurons

#### Specification and Fate

dA2 subpopulation, which appears ventral to dA1 group, exists only from r7 and caudally (Figure 1A). This excitatory subgroup is derived of Olig3^+^/Neurogenin1/2^+^ (Neurog1/2^+^) progenitors that upon maturation express the transcription factors Lhx1/5, Forkhead box protein 2 (Foxp2), and Pou4f1 (Figure 1C) (Landsberg et al., 2005; Storm et al., 2009). Notably, although this subgroup extends along the spinal cord as dI2, their express Foxd3 instead of FoxP2 (Storm et al., 2009). The mechanisms underlying this molecular difference as well as the appearance of dA2 INs only in the posterior RL, are currently unknown. Utilization of Wnt1-reporter mouse to lineage-trace RL-cell populations demonstrated a small contribution of r7-derived Neurog1^+^ neurons to the inferior olivary nucleus (ION), a major pre-cerebellar center that connects to the cerebellar Purkinje layer *via* climbing fiber (CF) axons (Landsberg et al., 2005). Yet, further fate map analyses are required to fully confirm dA2 fate as part of the ION, as dA4 subgroups were also suggested to assemble the ION, as will be described below.

#### dA2-Axonal Projections

The axonal projection patterns of dA2 neurons have not yet been delineated. This is in contrast to the spinal cord, where genetic labeling of dI2 INs in the chick spinal cord uncovered their contralateral ascending or descending projections, according to the thoracic/sacral level, before turning laterally in the white matter toward the LF (Avraham et al., 2009). Genetic lineage tracing of dA2 INs is needed to trace their axons and decipher whether they join the CF tract as well as possible other tracts in the developing brainstem.

### Class A: dA3 Interneurons

#### Specification and Fate

The dA3 subpopulation originates ventrally to the dA1 subgroup in r4–r7 (Figure 1A). Similar to other excitatory class A dINs, dA3 progenitors express Olig3 and Pou4f1, in addition to the Paired-like homeobox 2b (Phox2b), T-Cell Leukemia Homeobox 3 (Tlx3), and LIM homeobox protein 1-beta (Lmx1b). Moreover, they are the most dorsal subgroup that expresses the mammalian achaete-scute family bHLH transcription factor 1 (Ascl1^+^) (Figures 1B,C) (Qian et al., 2001; Sieber et al., 2007; Kim et al., 2008; Storm et al., 2009). Previous studies have suggested the role of Tlx3 in regulating the dA3-glutamatergic cell fate (Cheng et al., 2005; Chen et al., 2012). However, Tlx activity cannot be attributed to all excitatory dA/dB, as it is not expressed in other glutamatergic subgroups.

dA3 INs were found to contribute to various viscerosensory autonomic components in the hindbrain (Brunet and Pattyn, 2002; Qian et al., 2002; Dauger et al., 2003; D’Autréaux et al., 2011; Gray, 2013). dA3 (Phox2b^+^/Tlx3^+^/Ascl1^+^) neurons that emerge from r4–r7 contribute to the nucleus of the solitary tract (NTS), a major relay station for visceral sensory information regulating the activity of the cardiovascular, respiratory, vocalization, and digestive systems (Qian et al., 2001; Dauger et al., 2003; Pattyn et al., 2006; Sieber et al., 2007; Storm et al., 2009; Hernandez-Miranda et al., 2017; Gasparini et al., 2020). In addition, dA3 neurons deriving from r7–r8 contribute to the area postrema (AP) nucleus, a chemoreceptive center in the dorsal hindbrain that responds to toxins *via* chemically induced vomiting, as well as to A5/A7 noradrenergic clusters of the lateral tegmental area of the pons, which are suggested to be involved in vasomotor and respiratory activities, as well as in transmitting noradrenergic inputs to the spinal cord. Finally, dA3 INs were also suggested to give rise to the non-tyrosine hydroxylase expressing neurons of the intermediate reticular formation at the rostral medullary levels, implicating their additional involvement in secondary viscerosensory processing (Anderson et al., 1997; Qian et al., 2001; Kang et al., 2007; Gray, 2013). Interestingly, Phox2b is indispensable for the viscerosensory fate of dA3, since in its absence visceral sensory neurons resemble dB3 somatic sensory neurons, which express Tlx3 and Ascl1 (like dA3) but are devoid of Phox2b (D’Autréaux et al., 2011).

#### dA3 Axonal Projections

**FIGURE 3:**
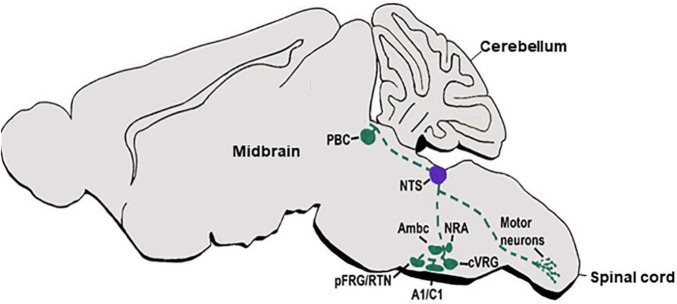
dA3 axonal projections. A schematic sagittal section of a main viscerosensory autonomic axonal circuit of the dA3 subclass. dA3-derived NTS nuclei (purple circle) are shown to project multiple axonal trajectories (green broken lines) to various target sites in the hindbrain or spinal cord (green circles). NTS, nucleus tractus solitaries; Pfrg/RTN, parafacial respiratory group/reticulotegmental nucleus; Ambc, ambiguous nucleus, compact part; cVRG, caudal ventral respiratory group; NRA, nucleus retro ambiguous; PBC, parabrachial.

In concordance with their homing at different autonomic centers, dA3-INs extend multiple axonal trajectories (Figure 3). Most studies traced projections of mature nuclei in adult brains. For example, projections from the NTS were examined in adult rodent brains by multiple antero/retrograde labeling approaches, revealing complex projections that ascend in ipsi/contralateral ventral/dorsal paths to innervate different sub-nuclear sites in the parabrachial nucleus (PBN), RTN, rostral ventrolateral medullary nucleus as well as within the respiratory compartments of the rostroventral respiratory group, preBötC and BötC nuclei (Herbert et al., 1990; Williams et al., 1996; Cunningham and Sawchenko, 2000; Karimnamazi et al., 2002; Alheid et al., 2011; Fu et al., 2019). Recently, a subpopulation of aldosterone-sensitive neurons, which express the dA3 markers Phox2B/Lmx1b, were also found to localize in the NTS and to control sodium appetite by projecting anteriorly to the PBN and pre-locus coeruleus (pLC) complex in the prepontine hindbrain (r1) as well as to the bed nucleus of the stria terminalis in the forebrain (Gasparini et al., 2018). Other NTS axons project ventrally and caudally to converge into the anterolateral funiculus toward different segments of the cervical and thoracic spinal cord, where axonal processes diverge and enter the ventral horn to innervate pre-motor neurons (Norgren, 1978; Mtui et al., 1993). Another ipsilateral projection elongates from the NTS through the reticular formation to terminate at the facial motor nucleus (Norgren, 1978). Finally, the NTS projects bilaterally to the hypoglossal nucleus, by crossing the midline and extending over the dorsomedial reticular formation toward the hypoglossal nucleus (Norgren, 1978). Notably, although advanced combinations of axonal labeling in knockout or reporter mice lines enabled to link Phox2b^+^ neurons in the NTS with these circuits (Qian et al., 2001; Dauger et al., 2003; Pattyn et al., 2006; Sieber et al., 2007; Smith et al., 2009; Storm et al., 2009; Hernandez-Miranda et al., 2017), tracking the gradual axonal growth of distinct dA3 INs before and after populating different subdomains along the NTS is missing. Unraveling this issue is particularly important since the NTS contains diverse intermingled subpopulations of neurons that modulate distinct functions by their extensive projections.

Anterograde axonal and retrograde cell body tract-tracing methods in the AP nucleus of the adult rat demonstrated its connectivity to a variety of different nuclei in both the medulla and upper brain regions. AP projections target the adjacent NTS and the PBN, as well as the ambiguous (AMB) nucleus, the dorsal motor nucleus of the vagus, dorsal regions of the tegmental nucleus, the cerebellar vermis, the paratrigeminal nucleus, the ventrolateral catecholaminergic column in the medulla, and the spinal trigeminal tract (van der Kooy and Koda, 1983; Shapiro and Miselis, 1985; Price et al., 2008; Stein and Loewy, 2010). Complex axonal trajectories extend from the AP to target these nuclei sites. For instance, ipsilateral AP axons extend either caudally or rostrally to target the NTS, or project in a rostroventral direction around the solitary tract and turn ventrolaterally *via* the reticular formation to either target the AMB or to further bifurcate and extend dorsorostrally toward the PBN and the pLC. Another AP tract joins the ventral spinocerebellar tract to enter the cerebellum, or projects dorsorostromedially and divides further to either terminate at the PBN or elongate further and terminate at the mesencephalic trigeminal nucleus (Shapiro and Miselis, 1985). Notably, no data exist regarding the development of these tracts in the embryo.

Retrograde and anterograde axonal tracing of the noradrenergic A5/A7 clusters in the adult rodent brain has demonstrated innervations to multiple midbrain regions of dopamine neurons (i.e., retrorubral field, ventral tegmental area, substantia nigra, interfascicular nucleus, ipsilateral rostral/central linear, and nuclei) (Mejías-Aponte et al., 2009). The A5 cell group was also found to project to the central nucleus of the amygdala, perifornical and dorsal areas of the hypothalamus, paraventricular nucleus of the thalamus, and the bed nucleus of the stria terminalis. This cluster also projects to PBN, the NTS, and the ventrolateral reticular formation of the medulla (Byrum and Guyenet, 1987). Further traditional or genetic axonal-labeling approaches had enabled the tracing of spinal projections of A5/A7 nuclei in adult rodent or cat brains, revealing descending projections *via* the ipsi/contralateral ventral and lateral funiculi. These projections further branch in a complementary fashion to reach the dorsal/ventral horns of the spinal cord at different axial levels (Fritschy and Grzanna, 1990; Clark and Proudfit, 1993; Bruinstroop et al., 2012). Finally, the non-tyrosine hydroxylase expressing neurons of the intermediate reticular formation in the medulla were suggested to relay cortical input to gustatory centers at the NTS and the PBN (Kang et al., 2007; Gray, 2013).

Altogether, dA3 INs exhibit complex fates and display multiple ascending and descending axonal routes (Figure 3), emphasizing their important contribution to various autonomic circuits in the CNS. Yet, knowledge is still missing regarding the developmental mechanisms that drive the different fates of dA3 subpopulations in individual rhombomeres. Further genetic-lineage tracing experiments of dA3 INs are also in need to fully determine the precise contribution of dA3 INs to the extensive axonal tracts described above Notably, one of the dA3 markers, Lmx1b, was recently shown to be necessary for controlling axonal growth of serotonergic 5-HT neurons in the hindbrain and dopaminergic circuits in the midbrain (Chabrat et al., 2017; Donovan et al., 2019), raising the possibility that Lmx1b may also affect dA3 axonal growth decisions.

### Class A: dA4 Interneurons

#### Specification and Fate

The dA4 population is the most ventral one amongst class A dINs (Figure 1A). Fate mapping experiments in chick and mice revealed that they derive from Pou4f1^+^/Olig3^+^/Mash^+^ progenitors that also express the bHLH factor Pancreas Specific Transcription factor 1a (Ptf1a) (Figures 1B,C) (Fedtsova and Turner, 1995; Sieber et al., 2007; Yamada et al., 2007; Storm et al., 2009; Hidalgo-Sánchez et al., 2012). dA4 neuronal group is the only excitatory subgroup that expresses Ptf1a (Yamada et al., 2007; Storm et al., 2009; Gray, 2013), like all other Ptf1^+^ dINs in the hindbrain or spinal cord are inhibitory (GABAergic/Glycinergic) and were suggested to depend on Ptf1a for their inhibitory neuronal fate (Glasgow et al., 2005; Hoshino et al., 2005). While the molecular profile of dA4 cells is uniform from r2 to r6, in r7 this subgroup also expresses Foxd3 (Sieber et al., 2007; Yamada et al., 2007; Storm et al., 2009; Iskusnykh et al., 2016). So far, the fate of dA4 neurons from r2–r6 is not clear. At variance, dA4 precursors originating from r7 to r11 were suggested to contribute to the ION, which is positioned in the caudal-ventral brainstem and coordinates signals to and from the cerebellar Purkinje cell layer to regulate motor coordination and learning (Sieber et al., 2007; Yamada et al., 2007; Zijing et al., 2008; Storm et al., 2009; Iskusnykh et al., 2016; Watson et al., 2019). Formation of ION is missing in Ptf1a-null zebrafish or mouse embryos, where Ptf1a^–/–^ cells shifted their fate to become MF neurons (Yamada et al., 2007; Itoh et al., 2020). Intriguingly, as Ptf1a or Ascl1 proteins are not only expressed in dA4 neurons but also their flanking dB1/dA3 subpopulations, further enhancer-intersection based approaches are required to distinctly map dA4 precursors at different AP levels, rather than their co-labeling with neighboring neuronal groups.

#### Axonal Projection Patterns

**FIGURE 4:**
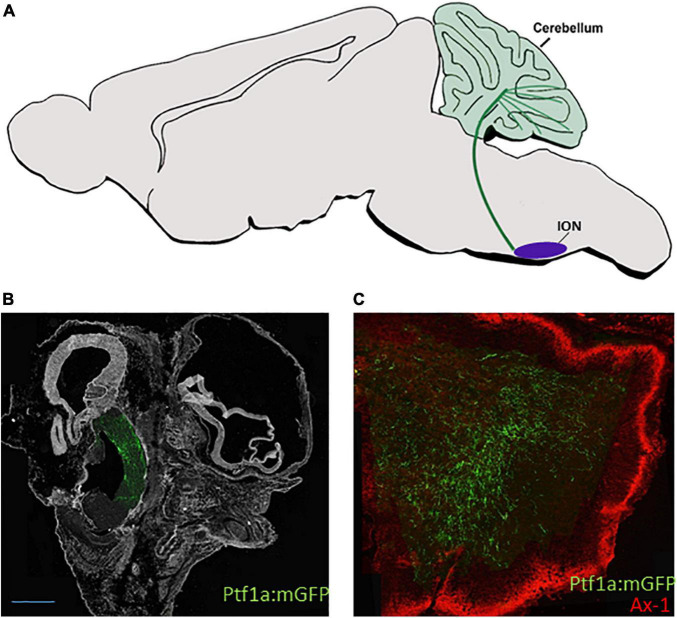
dA4 axonal projections. **(A)** A schematic sagittal section of the climbing fiber (CF) circuit of the dA4 subclass. dA4-derived ION (purple circle) is shown to project axonal trajectory (green line) to the Purkinje cell layer of the cerebellum (green circles). **(B,C)** Sagital sections from E9.5 **(B)** or E13.5 **(C)** chick embryos that were electroporated at E2.5 with a Ptf1a enhancer:Cre based plasmid together with lox-membranal GFP-lox plasmid (ptf1a:mGFP) to specifically label dA4 neurons at r7. The section in **(B)** shows dA4-derived axonal trajectories ascending from the caudal hindbrain toward the developing cerebellum. The section in **(C)** shows dA4-derived axonal trajectories that terminate in the cerebellum. An outer granular layer of the cerebellum is marked by Axonin 1. Bars, 50 μm. ION, inferior olivary nuclei; Ax-1, axonin 1.

Climbing fiber axons were traced by traditional retrograde/anterograde approaches in all vertebrates and found to originate from the caudal and rostral ION (Altman and Bayer, 1987b; Paradies and Eisenman, 1993; Sawada et al., 2008; Reeber et al., 2013). While all ION axons cross the FP and grow in the dorsomedial inferior cerebellar peduncle toward the contralateral cerebellum, those originating from the caudal ION, which lies in crypto-rhombomeres r10, r11, project to the posterior cerebellum whereas those deriving from more rostral ION position (r8 and r9) enter the cerebellum through the lateral inferior cerebellar peduncle, also termed the restiform body, turn to a dorsolateral route and innervate the lower strata of the embryonic Purkinje cell multilayer. Fate map studies of Ptf1a^+^ neurons in wild type and mutated zebrafish/mouse embryos confirmed that Ptf1a^+^ ION neurons extend excitatory commissural projections that innervate Purkinje cells (Figure 4A) (Yamada et al., 2007; Bae et al., 2009; Hashimoto and Hibi, 2012; Itoh et al., 2020). However, a direct link between projections of lineage-traced dA4 neurons from different ION subdomains to different cerebellar lobules is still missing. Moreover, as most studies traced this circuit at stages following ION formation, the gradual growth of dB4/Ptf1a^+^ axons from soon after their differentiation in the RL remained elusive. Using an enhancer-based conditional expression system in the chick embryo combined with the Ptf1a enhancer element, we have targeted PTF1a^+^ precursors exclusively at r7 and demonstrated that their axonal crossing and growth toward the cerebellum (Figures 4B,C) is initiated much before their neuronal soma migrate to the ION and establish the mature olivocerebellar circuit (Meredith et al., 2009; Kohl et al., 2015).

### Class B: dB1 Interneurons

#### Specification and Fate

The dB1 subpopulation is the dorsal most group amongst class B dINs, ventrally flanking dA4 (Figure 1A). This inhibitory group expresses a combination of molecular markers including Ptf1a and Ascl1 in their progenitorial stage, followed by upregulation of Lbx1, Lhx1, Lhx5, and Pax2 (Figures 1B,C) (Gross et al., 2002; Müller et al., 2002; Glasgow et al., 2005; Sieber et al., 2007; Fujiyama et al., 2009; Storm et al., 2009; Hoshino, 2012; Kohl et al., 2015; Nothwang, 2016). Multiple lineages tracing studies in mice and chicks, together with the generation of Ptf1a^–/–^ mice, have indicated that dB1 neurons migrate to various locations in the hindbrain and contribute inhibitory outputs to multiple nuclei centers, according to their rhombomeric origin (Sieber et al., 2007; Tashiro et al., 2007; Hori and Hoshino, 2012; Iskusnykh et al., 2016). For instance, dB1/Ptf1a^+^ neurons from r2–r5 were found to contribute to the auditory system by settling in the DCN, or the avian homolog nucleus angularis (NA) (Farago et al., 2006; Fujiyama et al., 2009; Kohl et al., 2015; Lipovsek and Wingate, 2018). The DCN, which receives inputs from the auditory nerve as well as from various brain sources, has a complex layered organization that resembles the cerebellum (Soares et al., 2002; Sawtell and Bell, 2013; Trussell and Oertel, 2018). Within the DCN, dB1 (Ptf1a^+^/lbx1^+^) derivatives were shown to give rise to multiple GABAergic neuronal cell types such as the inhibitory stellate cells, cartwheel cells, and Golgi cells, as well as to a small glycinergic population within the VCN (Farago et al., 2006; Fujiyama et al., 2009; Schinzel et al., 2021). Moreover, comparative analyses of the origin of different vestibular nuclei in mice and chick embryos have indicated that dB1/Ptf1a^+^ neurons from r2–r8 also contribute to the medial, lateral, and descending vestibular nuclei (MVN/LVN/DVN), three relay sensory hubs located in the medial column of the medulla and function to control eye, head and neck movements to maintain balance (Marín and Puelles, 1995; Díaz et al., 2003; Maklad and Fritzsch, 2003; Pasqualetti et al., 2007; Yamada et al., 2007; Straka et al., 2014; Kohl et al., 2015; Lipovsek and Wingate, 2018; Díaz and Puelles, 2019; Lunde et al., 2019), as well as to the spinal trigeminal nucleus and solitary nucleus (Yamada et al., 2007). This wealth of derivatives raises the question as to how distinct lineages of dB1 inhibitory neurons are being generated from the longitudinal Ptf1a^+^ progenitorial domain in the hindbrain RL.

#### Axonal Projection Patterns

**FIGURE 5:**
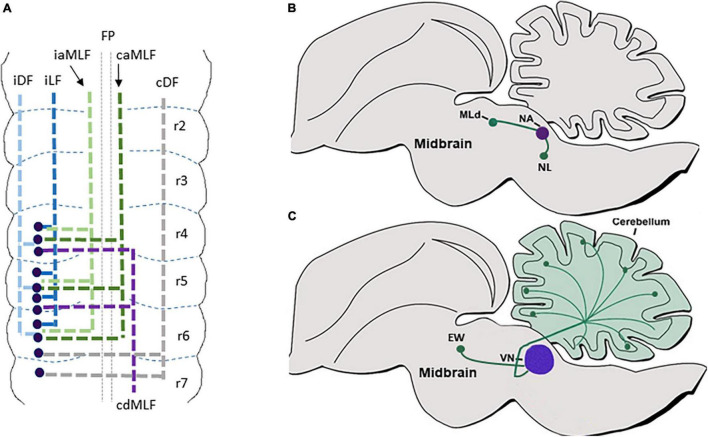
dB1 axonal projections. **(A)** A summary of dB1 axonal projections as seen in a flat-mount view of E6.5 chick embryonic hindbrain. dB1 cell bodies are shown as blue dots. Each axonal tract is shown in a different color and refers to a distinct funiculus. **(B)** A schematic sagittal section of the main auditory axonal circuits of dB1 subclass in the chick hindbrain. dAB1-derived NA (purple circle) is shown to project axons (green lines) to the NL in the medulla or to the MLd in the midbrain (green circles). **(C)** A schematic sagittal section of the main vestibular axonal circuits of dB1 subclass in the chick hindbrain. dA1-derived VN (purple circle) is shown to project axons (green lines) to the cerebellum and EW nuclei in the midbrain (green circles). FP, floor plate; iDF, ipsi dorsal funiculus; iLF, ipsilateral funiculus; iaMLF; ipsilateral ascending; caMLF, contralateral ascending medial longitudinal funiculus; cdMLF, contralateral descending medial longitude in nucleus laminaris; MLD, mesencephalicus lateralis pars dorsalis; VN, vestibular nuclei; EW, Edinger–Westphal.

Multiple axonal labeling studies and genetic fate maps uncovered the projections of the auditory and vestibular centers, which are likely to contain dB1/Ptf1a^+^ neuronal derivatives. For instance, the mammalian DCN/avian NA was found to form local medullary connections with the ipsilateral mammalian VCN/avian NM. This center projects to the nearby SON and the lateral lemniscal nuclei and extends projections through the ipsi and contralateral DF into the mammalian inferior colliculi/avian nucleus mesencephalicus lateralis pars dorsalis (MLD) in the anterior midbrain, an auditory center that transmits inputs to the medial geniculate body of the thalamus (Figure 5B) (Rubel and Parks, 1975; Takahashi and Konishi, 1988; Puelles et al., 1994; Cant and Benson, 2008; Krützfeldt et al., 2010a,b; Trussell and Oertel, 2018). Moreover, the different vestibular nuclei project *via* multiple ipsi and contralateral tracts to either descend to the spinal cord *via* the lateral or medial vestibulospinal tract or ascend *via* the medial longitudinal fascicle (MLF) to the midbrain Edinger–Westphal nuclei (EW, an autonomic parasympathetic component of the oculomotor nuclear complex that connects to the orbit ciliary ganglion) (Figure 5C). Other vestibular neurons form local connections between different vestibular nuclei or ascend to the cerebellum *via* vestibulocerebellar mossy fibers (Figure 5C) (Akert et al., 1980; Díaz et al., 1998; Straka et al., 2001, 2014; Balaban, 2003; Barmack, 2003; Pasqualetti et al., 2007; Gottesman-Davis and Peusner, 2010; Chagnaud et al., 2017; Di Bonito et al., 2017; Lunde et al., 2019; Ando et al., 2020). Albeit the importance of these brainstem circuits, the association of these multiple axonal projections to the dB1 subgroup was not fully confirmed, as neurons within different vestibular nuclei were found to derive from additional dINs such as dA1 and dB2 subgroups.

To address this issue, we aimed at tracing the axons of dB1 dINs, from soon after their birth up to their arrival at their target sites. By electroporating Ptf1a-enhancer element upstream to Cre recombinase along with conditional GFP reporter plasmids we were able to reliably label dB1(Ptf1A^+^/Lhx1/5^+^/Pax2+) dINs and demonstrate their multiple axonal projections that extended at subsequent time points (Figure 5A) (Meredith et al., 2009; Kohl et al., 2015). The first-appearing axons crossed the floor-plate and turned rostrally, joining either the contralateral MLF or the contralateral DF. Next, an ascending ipsilateral axonal tract began to project along with the ipsilateral MLF. Finally, two more ascending ipsilateral projections were evident; one emerged from a medial position forming an ipsilateral funiculus (LF) whereas the other elongated in a dorsal position forming an ipsi dorsal funiculus (DF) (Figure 5A). These axons projected and terminated in the medulla, cerebellum, MLD, and EW nuclei, invariably with the above-mentioned axonal routes of the vestibular and auditory nuclei (Figures 5B,C). These findings enabled us to connect the dB1 lineage with typical hindbrain tracts and target sites, as well as to uncover that dB1 axons begin to project toward different targets before their cell body migrate and settle in their final VN/CN centers (Kohl et al., 2015).

Taken together, the inhibitory dB1/Ptf1a^+^ subclass contributes to various types of brainstem nuclei, projects into discrete tracts at different time points, and synapses at multiple target sites. Their various fates and functions indicate that the dB1 neuronal group is likely to be composed of several subpopulations, each with its birth-time and fate. Intriguingly, since a single rhombomere gives rise to different dB1 axonal tracts and to several neuronal lineages that settle at various nuclei centers, it is likely that several rhombomere-specific regulators, which are unknown yet, act in defined spatiotemporal patterns to provide such intra-segmental diversity.

### Class B: dB2 Interneurons

#### Specification and Fate

The dB2 subpopulation is an excitatory group that develops in r2–r6, ventral to dB1 (Figures 1A,B). In contrast to its neighboring subgroups, it does not express Ascl1 in its progenitorial state but expresses Lbx1 and Phox2b upon differentiation (Figure 1B). In r4–r6, dB2 INs also express Phox2a, indicating at least two dB2 subgroups in the hindbrain (Sieber et al., 2007; Dubreuil et al., 2009; Rose et al., 2009a,b; Storm et al., 2009). Interestingly, this subpopulation is hindbrain-specific as no equivalent spinal dIN subgroup exists. Genetic fate map studies in rodents, combined with the generation of Lbx1/Phox2b-deficient mice, uncovered the contribution of dB2 (Lbx1^+^/Phox2b^+^) neurons to the RTN/parafacial respiratory group (pFRG) in the ventral medulla. This glutamatergic center relays modulatory input to the preBötC to control respiration rhythm by chemosensing CO_2_ levels in the blood (Stornetta et al., 2006; Pagliardini et al., 2008; Dubreuil et al., 2009; Guyenet et al., 2009; Thoby-Brisson et al., 2009; Feldman and Kam, 2015; Ikeda et al., 2015, 2017). Notably, the dB2-RTN precursors begin to express Atoh1 once they mature, serving as the only non-dA1 subgroup which requires this gene for its development (Dubreuil et al., 2009; Rose et al., 2009a,b; van der Heijden and Zoghbi, 2018). Elegant lineage tracing strategies uncovered that dB2/Phox2b^+^ RTN neurons arise from r3/r5/Krox20^+^ domains, and as such, mouse or human mutations in Phox2b or Krox20 lead to respiratory rhythm impairments (Jacquin et al., 1996; Weese-Mayer et al., 2005; Dubreuil et al., 2008; Pagliardini et al., 2008; Champagnat et al., 2009; Thoby-Brisson et al., 2009). In addition, dB2 neurons are also likely to contribute to the VN complex; fate map analysis of Hoxb1^GFP^ reporter mice showed that the LVN, which regulates the vestibulospinal reflex to maintain proper balance *via* the back and limb muscles, originate from Hoxb1^+^ precursor neurons in r4 which co-express the dB2 markers Lbx1/Phox2b/Phox2a, and fails to form in Hoxb1-null mice (Díaz et al., 1998; Maklad and Fritzsch, 2003; Chen et al., 2012; Di Bonito et al., 2015). Yet, although detailed chick/mouse fate map studies have previously delineated the rhombomeric origin of all VN subtypes in the hindbrain, (Díaz et al., 1998; Cambronero and Puelles, 2000; Pasqualetti et al., 2007), their association to dB2 dINS that originate from various rhombomeres has not been shown yet.

#### Axonal Projection Patterns

**FIGURE 6:**
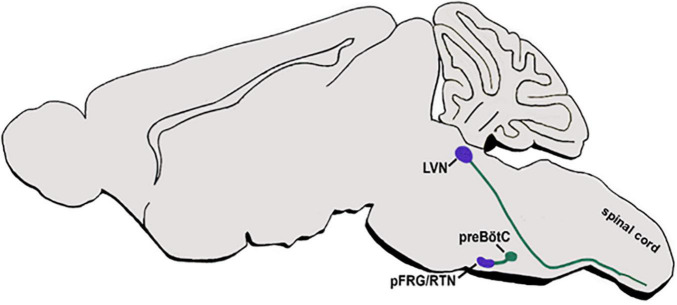
dB2 axonal projections. A schematic sagittal section of the main respiratory and vestibular circuits of dB2 subclass. dB2-derived pFRG or LVN nuclei (purple circles) are shown to project axons (green lines) to the hindbrain preBötC nuclei or to descend toward the spinal cord motor neurons (green circles). pFRG/RTN, parafacial respiratory group/reticulotegmental nucleus; LVN-lateral vestibular nucleus; preBötC-pre Bötcinger.

As described above, intersectional fate maps demonstrated the contribution of dB2 dINs to respiratory and vestibular circuits. However, knowledge regarding the axonal patterns of genetically-identified dB2 dINs, either before their arrival to their nuclei centers or after they settle in their final destinations, is sketchy. Data from Atoh1^lacZ^ reporter mice have demonstrated that mature pFRG/RTN neurons extend LacZ-labeled axons toward the ipsilateral preBötC (Figure 6) (Huang et al., 2012). Furthermore, multiple anterograde/retrograde studies, as well as electrophysiological analyses in the mature brainstem, have demonstrated that the pFRG/RTN relays glutamatergic inputs to other hindbrain areas, in addition to the pre-BötzC centers, such as to the ventral respiratory column in the medulla, the ipsilateral, ventrolateral, and intermediate subnuclei of the NTS, the PBN/KF at the dorsolateral pons, and the noradrenergic A5 cluster (Stornetta et al., 2006; Dubreuil et al., 2009; Guyenet et al., 2009; Thoby-Brisson et al., 2009; Bochorishvili et al., 2012; Feldman and Kam, 2015; Ikeda et al., 2017). Despite these findings, it is not fully clear whether all these projections arise from dB2-derived neurons in the pFRG/RTN or from other types of neurons that cluster in these heterogenic nuclei.

In addition, r4-derived VLN neurons, which are likely to originate from dB2 subpopulation, were found to project to the ipsilateral vestibulospinal tract (LVST), that descends along the spinal cord to connect to motor neurons of the extensor musculature of the limbs and the trunk (Figure 6). These LVST axons extend in a ventromedial direction toward the inferior olive, thereupon turning and descending through the medullary reticular formation within the ipsilateral ventral funiculus, and terminates at cervical and lumbosacral levels on INs residing in the ventral gray column (Díaz et al., 1998; Auclair et al., 1999; Straka et al., 2001, 2014; Maklad and Fritzsch, 2003; Pasqualetti et al., 2007; Chen et al., 2012; Liang et al., 2014; Di Bonito et al., 2015; Lunde et al., 2019).

Altogether, the complex reality of several dB2 subgroups as recognized by their unique transcriptional profile and fates among different rhombomeres, require further fate map investigations to reveal their precise destination and axonal connection to different somatosensory brain circuits.

### Class B: dB3 Interneurons

#### Specification and Fate

The excitatory dB3 neuronal population is born ventrally to the dB2 subgroup in r2–r6, and to dB1 in r7 (Figure 1A). dB3 is derived of a Ascl1^+^ progenitor domain and has a unique transcriptional profile of Lbx1/Tlx3/Lmx1b/Pou4f1/FoxP2 (Cheng et al., 2004; Mizuguchi et al., 2006; Sieber et al., 2007; Pagliardini et al., 2008; Storm et al., 2009; D’Autréaux et al., 2011; Gray, 2013) (Figures 1B,C). Multiple fate map studies of Ascl1^+^/Lbx1^+^/Tlx3^+^ neurons, as well as knockout of these genes, have suggested that Ascl1^+^/Lbx1^+^/Tlx3^+^ neurons contribute to the development of somatic sensory nuclei in the hindbrain, such as the spinal trigeminal nuclei (Sp5) and the principal trigeminal nucleus (Pr5), that relay various sensory modalities including temperature, touch, and pain from the ipsilateral faces (Qian et al., 2001, 2002; Sieber et al., 2007; Kim et al., 2008). Yet, genetic intersection approaches to target only dB3 dINs, rather than various Ascl1^+^/Tlx3^+^/Lbx1^+^-expressing neurons, have not been performed. Hence, the involvement of this subgroup in the brainstem somatic sensory system as well as its possible contribution to other hindbrain nuclei centers needs to be fully determined.

#### Axonal Projection Patterns

Axonal projections of brainstem relay somatic sensory neurons have been well described in adult brains. For instance, neurons originating from different subparts of the Sp5 nucleus project to several thalamic domains *via* the contralateral ventral trigeminal tract, such as the ventral posteromedial nucleus, the posterior group, and the region intercalated between the anterior pretectal and the medial geniculate nuclei. Sp5 neurons were also shown to project to the contralateral inferior colliculus in the midbrain, as well as to the local Pr5 nuclei and the granular, bushy, and stellate cell layers of the brainstem CN (Veinante et al., 2000; Zhou and Shore, 2006; Zeng et al., 2011; Heeringa et al., 2018). Furthermore, different subsets of Pr5 neurons were shown to project to centers in the medulla and pons such as the ipsi and contralateral solitary tract, the rostroventrolateral reticular nucleus, the AMB nucleus, the lateral reticular nucleus, and the ipsilateral PBN, as well as to the red nucleus (de Sousa Buck et al., 2001; Pinto et al., 2007). Interestingly, Lbx1^–/–^ mice were reported to extend misrouted tracts, where medullary longitudinal fibers shifted from ventral to more dorsal positions (Pagliardini et al., 2008). Yet, whether these fibers extended from dB3/Lbx1^+^ neurons in the Sp5/Pr5 nuclei, is not fully clear. Moreover, as Sp5 and Pr5 nuclei are divided into several sub-centers, the relative contribution of dB3 neurons to these nuclei and their multiple axonal projections has to be deciphered.

### Class B, dB4 Interneurons

#### Specification and Fate

The dB4 subpopulation is the ventral most group of hindbrain dINs, located ventral to dB3 (Figure 1A). Derived from Neurog1/2 progenitor domain, dB4 INs are inhibitory and express a combination of markers including Lhx1/5, Pax2, bHLHb5, Wilms tumor protein (Wt1), and presumably also the double sex/male abnormal 3 (DMRT3) (Figures 1B,C) (Gross et al., 2002; Müller et al., 2002; Sieber et al., 2007; Gray, 2008; Pagliardini et al., 2008; Hernandez-Miranda et al., 2017; Schnerwitzki et al., 2020). Notably, their spinal cord homolog group dI6, which shares a similar molecular profile and DV localization as dB4, was found to consist of three distinct subgroups, based on their singular or co-expression of Wt1 and/or DMRT3 (Gross et al., 2002; Helms and Johnson, 2003; Vallstedt and Kullander, 2013; Hernandez-Miranda et al., 2017; Schnerwitzki et al., 2020). dI6 neurons were found to migrate to the ventral horn of the spinal cord and to coordinate locomotion in different mammals (Andersson et al., 2012; Vallstedt and Kullander, 2013; Haque et al., 2018; Schnerwitzki et al., 2018). Nevertheless, it is currently uncertain whether the dB4 subgroup is also heterogeneous in Wt1/DMRT3 expression and whether it also plays a role in locomotion coordination.

In a previous study, hindbrain WT^+^ neurons were discovered in the AP nucleus, suggesting that dB4/WT^+^ neurons may be fated to contribute an inhibitory module to this nucleus, in addition to the presence of excitatory dA3 neurons in the AP (Sharma et al., 1992). Recent data have uncovered an additional fate of Wt1^+^ neurons in the caudal ventral respiratory group (cVRG) (Schnerwitzki et al., 2020). This neuronal cluster is positioned in the caudal-most part of the respiratory column and is known to participate in the activation of motor neurons in the cervical spinal cord, which in turn innervate the diaphragm muscles, leading to their contraction and thereby to inspiration (Ezure et al., 2003; Alheid and McCrimmon, 2008). As such, ablation of Wt1^+^ neurons resulted in the death of neonates due to the inability to initiate respiration, suggesting a vital role for Wt1^+^ neurons in breathing (Schnerwitzki et al., 2020). Since WT expression in the dINs is restricted to the dB4 subgroup, these results strongly suggest the contribution of the dB4 subgroup to respiratory control. Yet, as dB4 dINs appear along the entire hindbrain AP axis, this subgroup is likely to contribute to additional brainstem nuclei.

#### Axonal Projection Patterns

**FIGURE 7:**
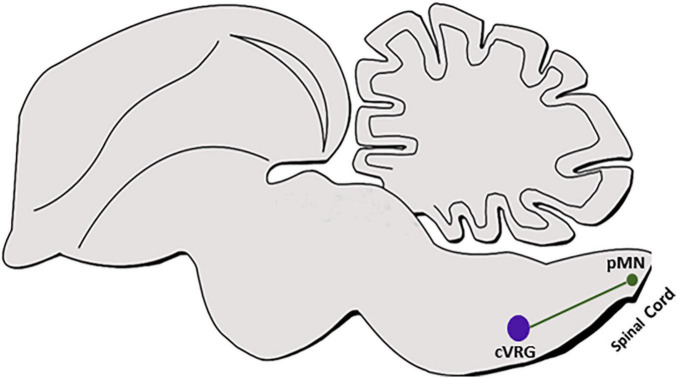
dB4 axonal projections. A schematic sagittal section of dB4/WT1^+^ axonal circuit. dB4-derived cVRG (purple circle) is shown to project axons (green line) to pMNs in the cervical spinal cord (green circle). WT, Wilm’s tumor 1; cVRG, caudal ventral respiratory group; pMN, pre motor neurons.

In the spinal cord, dI6/DMRT^+^ neurons project ipsi and contralateral axons that innervate somatic motoneurons of tibialis anterior and/or gastrocnemius (Andersson et al., 2012; Vallstedt and Kullander, 2013). Although dB4/WT^+^ subpopulation was found to contribute to the AP and cVRG (Figure 7) (Sharma et al., 1992; Schnerwitzki et al., 2020), genetic labeling of their axons and target sites has not been performed as of yet. Hence, while multiple ipsi and contralateral axonal projections are known to arise from the AP to target multiple nuclei sites in the medulla and the upper brain (van der Kooy and Koda, 1983; Shapiro and Miselis, 1985; Price et al., 2008; Stein and Loewy, 2010), it is not yet clear whether any of these targets are innervated by dB4 axons. In addition, cVRG sends commissural axons that descend in the ventromedial medulla toward their premotor neuronal targets in the contralateral cervical spinal cord that are responsible to activate inspiratory and expiratory motor neurons (Ezure et al., 2003; Alheid and McCrimmon, 2008). Although this tract is likely to be projected from the dB4/WT^+^ cVRG neurons (Figure 7) (Schnerwitzki et al., 2020), tracing of genetically labeled dB4 axons is required to fully support this data as well as to identify additional projections from more rostral dB4 neurons.

## Molecular Regulators That Control Axonal Growth of Hindbrain dINs

Axonal growth is a multi-event process that includes axonogenesis, pathfinding, arborization, and establishment of terminals on appropriate postsynaptic structures (Chédotal and Richards, 2010; Stoeckli, 2018; Comer et al., 2019). As different dA/dB subgroups display multiple types of axonal routes, they are likely to share common molecular cues that, for instance, guide their axonal crossing across the FP or extend their projections in fasciculated funiculi. In parallel, each neuronal subtype is also likely to respond to individual cues that determine its particular axonal pattern. Identification of such cues is crucial for uncovering how brainstem circuitries are assembled during normal development or misassembled in neurodevelopmental disorders. Less knowledge exists on dA/dB hindbrain axons, as opposed to their dI/dB spinal cord homologs. A summary of the knowledge on dA/dB axonal growth regulation is presented here, according to different types of molecules. Notably, although dINs undergo extensive neuronal cell body migration in parallel to growing axons, this topic is not discussed here.

### Transcription Factors

#### Lhx

Lim-HD proteins control multiple aspects of neuronal development, including axonal guidance (Hobert and Westphal, 2000; Kania et al., 2000; Shirasaki and Pfaff, 2003; Wilson et al., 2008; Avraham et al., 2009; Roy et al., 2012). In particular, different Lhx protein members are expressed in the spinal cord and forebrain and regulate axonal growth and neurotransmitter type of different neuronal populations (Pillai et al., 2007; Wilson et al., 2008; Avraham et al., 2009; Palmesino et al., 2010; Chou and Tole, 2019). In the hindbrain, particular dA/dB subgroups express different Lhx’s proteins; Lhx2/9 are specific to dA1, while Lhx1/5 are specific to dB1 and dB4 (Gray, 2008; Hernandez-Miranda et al., 2017). To address whether these factors govern the specific axonal paths of each subgroup, we have switched between the expression of the dA1-specific Lhx2/9 and the dB1-specific Lhx1/5 genes, such that each neuronal population was forced to express the Lhx’s of the other. This modification did not affect their specification but was sufficient to misdirect axonal patterns and target sites of one subgroup to phenocopy the patterns of the other (Kohl et al., 2012, 2015). As such, dA1 axons shifted to target the Purkinje cell layer, and dB1 axons terminated at the granular cell layer of the cerebellum, demonstrating the important regulatory role of the Lhx code in assembling dA/dB axonal circuits in the developing brainstem. To uncover the mechanism by which Lhx proteins control dA/dB axonal patterns, downstream effector genes that act as guidance cues should be uncovered. Notwithstanding is the fact that although Lhx2/9 or Lhx1/5 are longitudinally expressed in dA1/dB1 subgroups along with the entire hindbrain, several axonal trajectories extend from each subgroup (Figures 2, 5), indicating that other factors guide the growth of axons within each group, in addition to Lhx proteins.

#### Hox

As Ho*x* genes are fundamental for the segmental identity and patterning of rhombomeres, their involvement in regulating axonal projections of dINs that originate from individual rhombomeres has been suggested. Well-designed fate map analyses of individual rhombomeres using Hox-specific enhancers, together with the characterization of mutant mice/zebrafish, uncovered the role of Hox genes in governing vestibular, trigeminal, branchial, auditory, pre-cerebellar, and somatosensory nuclei projections (Carpenter et al., 1993; Marín and Puelles, 1995; Gavalas et al., 1997; Glover, 2000; del Toro et al., 2001; McClintock et al., 2002; Maklad and Fritzsch, 2003; Farago et al., 2006; Oury et al., 2006; Pasqualetti et al., 2007; Geisen et al., 2008; Narita and Rijli, 2009; Di Bonito et al., 2013, 2015; Lipovsek and Wingate, 2018; Beiriger et al., 2021). For example, Hoxa2 was found to be required for the contralateral projections of dA1-derived AVCN axons to the medial nucleus of the trapezoid body (MNTB), which aberrantly innervated the ipsilateral MNTB in Hoxa2 mutants (Di Bonito et al., 2013). Hoxa2 was also found to be involved in the topographic specificity of axons extending from the dB3-derived Pr5 nuclei to the thalamic ventral posterior medial (MPV) nucleus (Oury et al., 2006). Yet, many of these studies focused more on neuronal cell body migration rather than on axonal projections or did not associate defined projection patterns with genetically identified dA/dB subclasses. Hence, more data is required to further illuminate the role and manner of action of Hox genes in governing axonal growth decisions of particular dA/dB dorsal INs.

#### Zic

The transcription factor Zic1, a member of the Zinc Finger of the Cerebellum (Zic) family, is expressed in PGN neurons in the ventral hindbrain. Zic1 was found to drive axon laterality choice to the ipsilateral, rather than the contralateral pontocerebellar tract, by inhibiting axonal midline crossing (DiPietrantonio and Dymecki, 2009). PGN neurons belong to dA1/Atho1^+^ MF neuronal subgroup that arises in the caudal hindbrain. While Zic1 effectors that inhibit dA1 axonal crossing are not known, previous studies in the spinal cord or the upper brain have found that Eph receptors are induced by Zic proteins to activate the repulsive Eph-ephrin signaling cue (García-Frigola et al., 2008; Lee et al., 2008; Escalante et al., 2013). Future studies are required to reveal whether Zic1 upregulates Eph receptors in MF neurons and whether it impacts axonal growth decisions in additional neuronal subgroups along the rhombic lip that extend axons to both sides of the hindbrain.

### Axonal Guidance Cues

#### Robo/Slit

Roundabout (Robo) family of transmembrane receptors and their soluble ligands Slit are chemorepulsive cues fundamental for commissural axonal guidance in the CNS (Tessier-Lavigne and Goodman, 1996; Dickson and Gilestro, 2006). Mutations in human Robo genes were found to disrupt hindbrain axon crossing in patients with horizontal gaze palsy with progressive scoliosis (Jen et al., 2004). Multiple studies in mice have shown a conserved role of Robo/Slit signaling in guiding commissural axons in the hindbrain, before and after crossing the FP (Lee et al., 2001; Causeret et al., 2002; Bloch-Gallego et al., 2005; Howell et al., 2007; Tamada et al., 2008; Bouvier et al., 2010; Renier et al., 2010; Mirza et al., 2013; Friocourt et al., 2019). For instance, PCN/MF neurons and auditory AVCN neurons, that are both generated from the dA1/Atoh1^+^ group, require Robo/Slit signaling for axonal elongation from the dorsal rhombic lip toward the FP and for crossing the midline, which fails to occur in Robo3 mutant mice (Marillat et al., 2004; Renier et al., 2010). Similarly, ION/CF neurons, that originate from dA4/Ptf1a^+^ progenitors and extend contralateral fibers toward the cerebellum, require Robo3 signaling for their axonal crossing, as shown by their abnormal extension of ipsilateral processes in Robo3 deficient mice (Marillat et al., 2004). At variance, the knockout of other Robo/Slit members such as Robo1/2 or Slit1/2 did not impair the midline crossing of PCN axons, although it severely affected their cell body migration (Geisen et al., 2008). Interestingly, Robo1/3 expression was found to be induced by Lhx2/9 and to control axonal growth of thalamocortical and spinal neurons (Wilson et al., 2008; Chatterjee et al., 2012). These findings may suggest that the regulatory role of Lhx2/9 on dA1 axons (Kohl et al., 2012, 2015) is mediated by Robo3. Yet, an opposite role of Lhx2/9 to prevent the expression of Slit was recently found in retinal ganglion axons (Yang et al., 2020), indicating that the inductive or inhibitory role of Lhx2/9 on Robo/Slit signaling is context-dependent. Another interesting upstream regulator of Robo3 in the hindbrain is Hoxa2; In the dA1-derived AVCN, Robo3 expression was found to be induced by Hoxa2 and to be necessary to guide AVCN axons to project into the contralateral medulla (Di Bonito et al., 2013).

#### Adhesion Molecules

Cell adhesion molecules (CAMs) are instructive for axonal pathfinding, elongation, and fasciculation (Walsh and Doherty, 1997; Colman and Filbin, 1999; Hirano et al., 2003; Bloch-Gallego et al., 2005; Pollerberg et al., 2013). Several previous studies discovered various cadherin (Cdh) subtypes (i.e., N-Cdh, E-Cdh, Cdh6/8/11, and protocadherins7/10) that are expressed in dA/dB-derived nuclei, such as in the trigeminal, raphe, inferior olive, pre-cerebellar, and vestibular nuclei (Shimamura et al., 1992; Redies and Takeichi, 1993; Korematsu and Redies, 1997; Taniguchi et al., 2006; Neudert and Redies, 2008). Confirmation of their role in axonal guidance was shown in the LRN, ECN, and PN, which originate from dA1 dINs and require N-Cdh and Cdh11 for their soma and axonal migration toward the FP, or Cdh7 for their projection toward the cerebellum (Taniguchi et al., 2006; Kuwako et al., 2014). Interestingly, an interplay between Robo and N-Cdh was shown to guide spinal commissural axons of dI1/dI2 subgroups into longitudinal tracts that participate in the spinocerebellar projection (Sakai et al., 2012), raising the possibility that these cues also cooperate in hindbrain PCN/ION axonal migration. Two additional adhesion molecules, the NgCAM-related CAM Nr-CAM and its receptor TAG-1 were also found to be expressed in the caudal hindbrain; Nr-CAM is expressed in the dA4-derived ION neurons whereas Tag1 is restricted to the dA1-derived PCN (Backer et al., 2002). As Tag1 and Nr-CAM play crucial roles in commissural axon guidance across the spinal cord midline (Stoeckli and Landmesser, 1995; Lustig et al., 2001), it remains to be determined whether they play a similar role in hindbrain axons. Finally, nectin-like proteins (Necl1/3) were found to be involved in the regulation of commissural axonal trajectories in the anterior hindbrain, as their perturbation caused abnormal fasciculation in the form of failure to turn longitudinally at the contralateral side of the rat hindbrain (Okabe et al., 2004). Although not genetically identified, their position suggests they are likely to arise from the dA1 subgroup in r1.

#### Eph-Ephrin

Eph proteins, including the Eph receptors and their ligands ephrins, are fundamental for axonal guidance in the CNS (Cheng et al., 1995; Cramer and Gabriele, 2014; Klein and Kania, 2014; Milinkeviciute and Cramer, 2020). A series of elegant studies in the auditory hindbrain uncovered the role of EphA4/B2/B3 receptors in regulating axonal connectivity of the dA1-derived NM-NL binaural circuit in avian, or in the projection of the VCN nuclei, which consist of dA1 and dB1 neurons, to the contralateral MNTB in mice. Disruption in these receptors in chick or mouse embryos resulted in axonal misrouting and various targeting errors (Cramer et al., 2004, 2006; Huffman and Cramer, 2007; Hsieh et al., 2010; Abdul-latif et al., 2015). EphA-ephrinA interactions also play a role in guiding the turning point of axons that project from caudal vestibular neurons (cC-VC) once they cross the midline and extend longitudinally toward the cerebellum (Zhu et al., 2006). Although the genetic identity of these neurons was not shown in this study, their cell body position and axonal patterns suggest that they originate from the dA1 subgroup. Finally, EphA-ephrinA interaction was also suggested to guide ION axons that derive from the dA4 subgroup to their correct targets in the cerebellum (Nishida et al., 2002). Interestingly, data from the spinal cord suggested that limb innervation by lateral motor column neurons depends on EphA4, which is a downstream target of Lhx1 (Kania et al., 2000), raising the possibility that also in the hindbrain, Lhx proteins, which were found to control dA1/dB1 axonal projections (Kohl et al., 2012, 2015), are upstream to Eph-ephrin signaling.

#### Netrin/Deleted in Colorectal Cancer

Multiple pieces of evidence have shown the involvement of the chemoattractant molecule Netrin and its receptor Deleted in Colorectal Cancer (DCC) in hindbrain commissural axons (Keino-Masu et al., 1996; Guthrie, 1997; Bloch-Gallego et al., 2005). For instance, dA1-derived VCN axons, which express DCC, cross the midline to target the contralateral superior olivary complex (SOC). Yet, mice mutated for DCC lack VCN axonal outgrowth (Howell et al., 2007). Moreover, dA1-derived PCN, dA4-derived ION, or dB3-derived Pr5 trigeminal nuclei draw their axons to cross the midline and ascend in defined funiculi toward upper brain regions. Their projection toward the midline, crossing of the FP, and post-crossing trajectories was severely impaired in Netrin or DCC mutants (Yee et al., 1999; Alcantara et al., 2000; Causeret et al., 2002; de Diego et al., 2002; Mirza et al., 2013; Shoja-Taheri et al., 2015; Dominici et al., 2017). Moreover, recent studies uncovered that in the aforementioned knockouts, some of these nuclei project abnormal axons into the PNS along the trigeminal, auditory, and vagal nerve routes (Moreno-Bravo et al., 2018; Yung et al., 2018). Together, these data indicate that the netrin-DCC signaling system is required for establishing proper axonal projections of auditory, pre-cerebellar, and somatosensory hindbrain neurons together with maintaining a clear CNS-PNS boundary in this region.

#### Neuropilin/Semaphorin

The role of the neuropilin (Npn) receptors and their semaphorin (Sema) ligands in axonal growth is well known in various sensory and motor neural systems (Neufeld et al., 2002; Meléndez-Herrera and Varela-Echavarría, 2006; Pasterkamp, 2012). In the hindbrain, Nrp1 and Sema3A were found to form gradients across the projections of A1-derived pontine axons (Solowska et al., 2002), whereas Npn-1/2 were shown to be expressed in axons projecting from the NTS, which contain dA3-derived neurons (Corson et al., 2013). These spatiotemporal expression patterns indicate that the Npn and Sema families of axon guidance molecules are potential molecular regulators for dA1 and dA3 axonal trajectories. Furthermore, previous data from zebrafish embryos have shown that hindbrain axons that project along the MLF, require Sema3D to promote their fasciculation, which was mediated by the upregulation of the adhesion molecule L1 CAM (Wolman et al., 2007). As many sorts of axons, including those projecting from several dA/dB neuronal subtypes, ascend or descend along the MLF, Npn-Sema signaling may have a broad role in hindbrain axonal guidance.

### The RNA Binding Protein Fragile X Mental Retardation Protein

**FIGURE 8:**
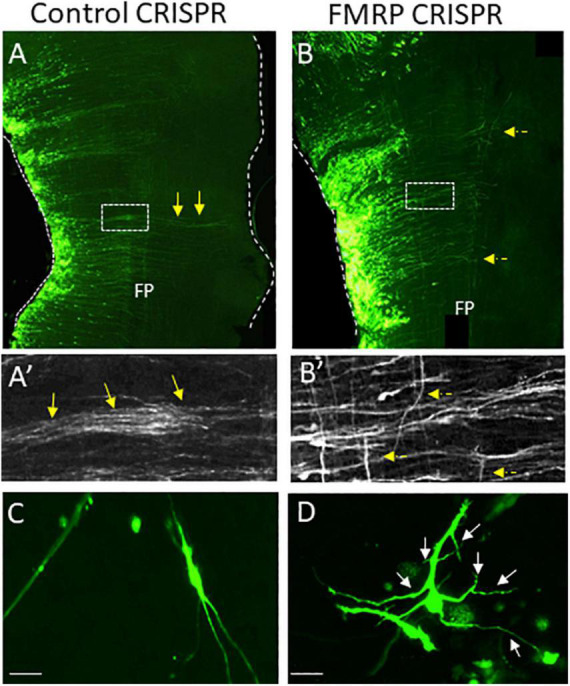
CRISPR-mediated FMRP knockout induces disoriented axonal growth in dA1-derived NM neurons in the chick hindbrain. **(A–B′)** Flat-mounted hindbrains from embryos electroporated with control **(A)** or FMRP **(B)** CRISPR/Cas9- guide RNA-GFP plasmids. Electroporated NM axons are GFP-labeled. Higher-magnification views of the boxed areas in **(A,B)** are shown in **(A′,B′)**. Yellow arrows indicate aligned axons that cross the hindbrain midline **(A,A′)**. Dashed yellow arrows indicate disoriented axons **(B,B′)**. **(C,D)** Cell cultures from GFP-expressing NM neurons that were electroporated with the above-mentioned plasmids. Control cells **(C)** project straight and oriented axons. FMRP-knockout cells **(D)** project over-branching axons Scale bars: 50 μm. FP, floor plate; NM, nucleus magnocellularis; CRISP, Crisper/Cas9-based plasmids.

The RNA binding protein FMRP is broadly expressed in the CNS where it acts as a reversible repressor of specific mRNA translation (Darnell et al., 2001; Davis and Broadie, 2017). Functional loss of FMRP leads to fragile X syndrome, a neurodevelopmental disorder with severe cognitive impairment (Hagerman et al., 2017). Multiple studies have supported the role of FMRP in axonal development, as, for example, FMRP knockout results in excessive axonal branches in motor neurons and abnormal projection patterns in the forebrain (Bureau et al., 2008; Shamay-Ramot et al., 2015; Scharkowski et al., 2018). Notably, FMRP was found to associate with RNAs that encode axonal guidance molecules, such as netrin and Dscam (Jain and Welshhans, 2016; Kang et al., 2019). We have recently uncovered the role of FMRP in dA1-derived neurons of the auditory NM nuclei in the chick. FMRP was found to localize in axons of dA1/NM neurons, and its downregulation led to perturbed axonal pathfinding, delay in midline crossing, excess branching of neurites, and axonal targeting errors during the period of auditory circuit development (Figure 8) (Wang et al., 2020). This finding provided the first *in vivo* identification of FMRP activity in developing axons in the hindbrain. Further studies are required to elucidate FMRP-downstream RNA targets in dA1 axons and to uncover whether fragile X patients suffer from axonal development deficits in the auditory brainstem.

## Concluding Remarks

The orderly and conserved development of the segmented hindbrain in vertebrates, together with its fundamental roles to transmit and process sensory-motor orders that arrive from the surroundings, or the spinal cord and higher brain centers, makes it a fascinating CNS domain. Yet, how it is set to produce diverse neurons that are programmed to assemble dedicated neuronal circuits that execute a wealth of physiological actions, is only partially clear. The ground plan to develop 8 dINs subclasses along the longitudinal axis of the hindbrain which have to respond to antagonistic roof plate and floor plate DV cues along with rhombomere-specific AP positional information is unique. Many important studies along the years have initially mapped definite DV and AP positions in the hindbrain that drive specific axonal tracts and neuronal clustering which in turn give rise to different brainstem nuclei. With the generation of transgenic and mutant animal models, the genetic code of all individual dA/dB subtypes has been decoded, enabling investigation of the developmental program, circuit formation, and fate of dA1-4/dB1-4 neuronal subgroups. Yet, many challenges remain to fully correlate the identity of specific dINs in the early embryo with brainstem nuclei and projections at advanced stages, to identify the mechanism by which each dA/dB subgroup, which displays a uniform genetic identity along the AP axis, can eventually give rise to different types of neuronal cell types with distinct axonal connections, target sites, and functions, and to uncover how multiple attractive and repulsive axonal cues are orchestrated to guide the step-by-step assembly of multiple axonal projections that arise from individual and/or neighboring dINs in the developing hindbrain.

## Data Availability Statement

The original contributions presented in the study are included in the article/supplementary material, further inquiries can be directed to the corresponding author.

## Author Contributions

DH, AK, YW, and DS-D participated in writing and revising the manuscript and preparing the figures and contributed to the data presented in the manuscript. All authors contributed to the article and approved the submitted version.

## Conflict of Interest

The authors declare that the research was conducted in the absence of any commercial or financial relationships that could be construed as a potential conflict of interest.

## Publisher’s Note

All claims expressed in this article are solely those of the authors and do not necessarily represent those of their affiliated organizations, or those of the publisher, the editors and the reviewers. Any product that may be evaluated in this article, or claim that may be made by its manufacturer, is not guaranteed or endorsed by the publisher.

## Abbreviations

AMB: ambiguous
AP: area postrema
AP: anterioposterior
Atoh1: atonal homolog 1
Ascl1: achaete-scute family bHLH transcription factor 1
AVCN: anteroventral cochlear nucleus
CAM: cell adhesion molecules
cC-VC: caudal vestibular neurons
Cdh: cadherins
CF: climbing fiber
CN: cochlear nuclei
CNS: central nervous system
cVRG: caudal ventral respiratory group
DCC: receptor Deleted in Colorectal Cancer
DCN: dorsal cochlear nuclei
g: dorsal funiculi
dINs: dorsal interneurons
DMRT3: double sex/male abnormal 3
DV: dorsoventral
DVN: descending vestibular nuclei
EW: Edinger–Westphal
FMRP: fragile X mental retardation protein
FoxB: forkhead box protein B
FP: floor plate
HD: homeodomain
INs: interneurons
ION: inferior olivary nucleus
ITR: intertrigeminal region
KF: Kölliker-Fuse
Lbx1: ladybird homeobox transcription factor
LF: lateral funiculi
LMX1a/b: LIM homeobox transcription factor 1 alpha/beta
LVN: lateral vestibular nuclei
LVST: lateral vestibulospinal tract
MF: mossy fiber
MLD: nucleus mesencephalicus lateralis pars dorsalis
MLF: medial longitudinal fascicle
MNTB: medial nucleus of trapezoid body
MPV: ventral posterior medial
MSO: medial superior olivary
MVN: medial vestibular nuclei
NA: nucleus angularis
Necl: nectin-like proteins
Neurog: neurogenin
NL: nucleus laminaris
NM: nucleus magnocellularis
Npn: neuropilin
NTS: nucleus tractus solitarius
PBN: parabrachial nucleus
PCN: precerebellar nuclei
pFRG: parafacial respiratory group
PGN: pontine gray nucleus
Phox2b: paired-like homeobox 2b
pLC: pre-locus coeruleus
PNS: peripheral nervous system
Pou4f1: POU domain class 4 transcription factor 1
Pou4f1: POU domain class 4 transcription factor 1
Pr5: principal trigeminal nucleus
preBötC: preBötzinger Complex
Ptf1a: pancreas-specific transcription factor 1a
r: rhombomere
RL: rhombic lip-
Robo: roundabout
RTN: retrotrapezoid nucleus
Sema: semaphorin
SOC: superior olivary complex
SON: superior olivary nuclei
Sp5: spinal trigeminal nuclei
Tlx: T cell leukemia homeobox
VCN: ventral cochlear nuclei
Wt1: Wilms tumor protein 1
Zic: zinc-finger of the cerebellum.
